# Supplementary material for: Systematic analysis of in-source modifications of primary metabolites during flow-injection time-of-flight mass spectrometry
Source: Anal Biochem. 2023 Mar 1;664:115036. doi: 10.1016/j.ab.2023.115036 (PMC9902335; doi:10.1016/j.ab.2023.115036)
Supplement: Multimedia component 1 [file mmc1.docx]

**Supplementary Data for:**

**Systematic analysis of in-source modifications of primary metabolites during flow-injection time-of-flight mass spectrometry**

Niklas Farke‡, Thorben Schramm‡, Andreas Verhülsdonk, Johanna Rapp, Hannes Link*

Bacterial Metabolomics, CMFI, University Tübingen, Auf der Morgenstelle 24, 7206 Tübingen, Germany

*corresponding author: hannes.link@uni-tuebingen.de

‡contributed equally

**Content:**

**Supplementary Data: Figure S1**: Ion peaks of nucleotides in negative ionization mode from fully labelled ^12^C- and ^13^C-labelled *E. coli* extracts.

**Supplementary Data: Figure S2**: Ion peak of reduced glutathione in negative ionization mode.

**Supplementary Data: Figure S3**: Boxplot showing the relative standard deviation (RSD) of the endogenous metabolites measured by FI-MS in positive and negative ionization mode.

**Supplementary Data: Figure S4**: Bar graphs showing the number of significant *m/z* features at different fragmentor/skimmer voltages and for LC-MS.


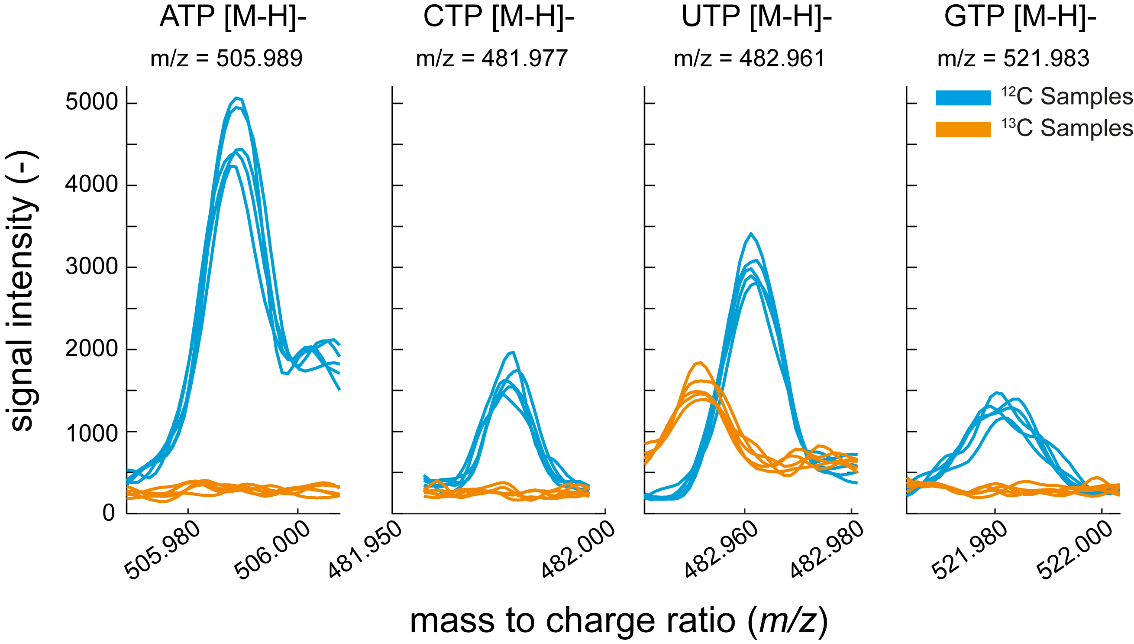


**Supplementary Data: Figure S1.** Ion peaks that are annotated to four nucleotides (ATP, CTP, UTP, GTP) in negative ionization mode. ^12^C-labelled E. coli samples are blue lines measured in five technical replicates. ^13^C-labelled E. coli samples are orange lines measured in five technical replicates. ATP, adenosine triphosphate; CTP, cytidine triphosphate; UTP, uridine triphosphate; GTP, guanosine triphosphate.


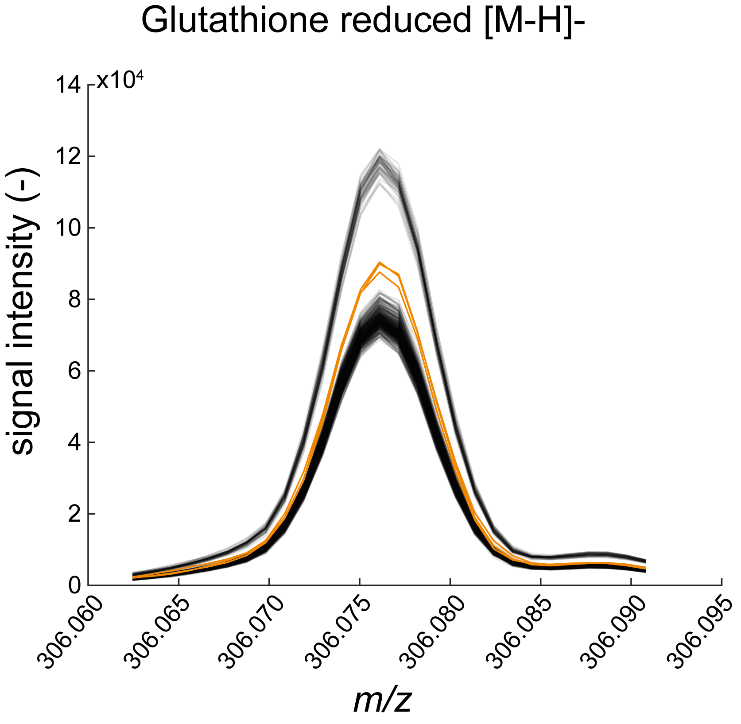


**Supplementary Data: Figure S2.** Ion peak of reduced glutathione (deprotonated) in negative ionization mode. The spike-in sample that contains reduced glutathione is indicated in orange, the other 159 spike-in samples are black.


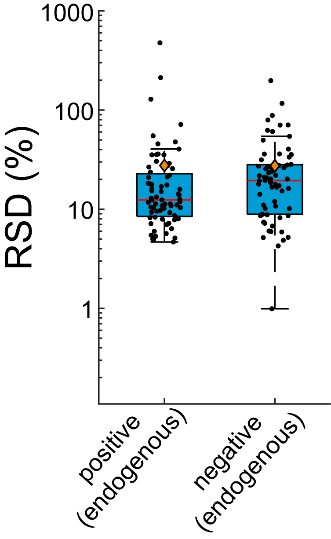


**Supplementary Data: Figure S3.** Boxplot showing the relative standard deviation (RSD) of the endogenous metabolites measured by FI-MS in positive and negative ionization mode. Black dots are the RSD for each endogenous metabolite. Upper and lower box edges indicate the 25 % and 75 % percentiles. The whiskers indicate the furthest point, at which samples were not considered as outliers. The red line indicates the median. Orange diamonds are the means.


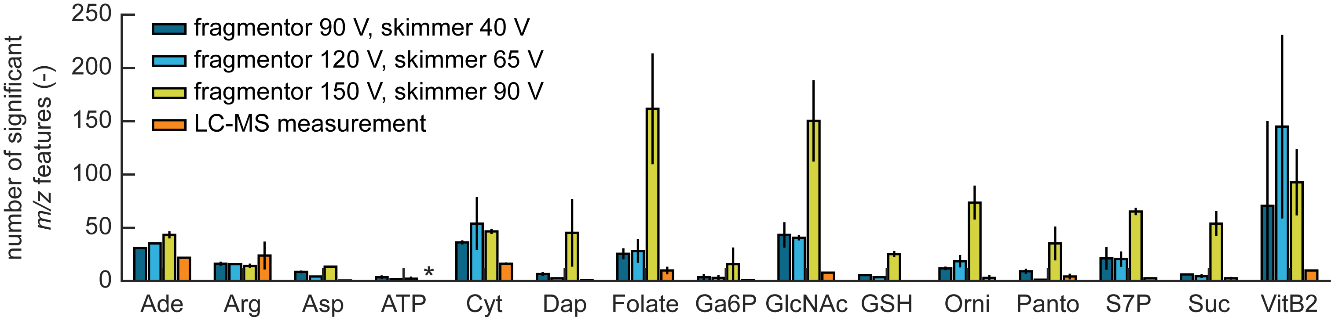


**Supplementary Data: Figure S4.** Bar graph showing the number of significant m/z features in spike-in samples (z-score > 3) measured by FI-MS at different fragmentor and skimmer voltages (dark blue 90/40 V, light blue 120/65 V, and yellow: 150/90V). The orange bar are samples measured by LC-MS using a HILIC column. *indicated that no peak was detected during LC-MS. Ade: Adenine, Arg: L-Arginine, Asp: L-Aspartate, ATP: Adenosine 5-triphosphate, Cyt: Cytidine, Dap, 2,6-Diaminopimelic acid, Ga6P: D-Glucosamine-6-phosphate, GlcNAc: N-Acetyl-D-glucosamine, GSH: L-Glutathione reduced, Orni: L-Ornithine, Panto: (R)Pantoate, S7P: Sedoheptulose-7-phosphate, Suc: Succinate, VitB2: riboflavin (vitamin B2).
